# The effects of absenteeism on nurses remaining on duty at a tertiary hospital of Limpopo province

**DOI:** 10.4102/curationis.v41i1.1924

**Published:** 2018-11-27

**Authors:** Masenyani O. Mbombi, Tebogo M. Mothiba, Rabelani N. Malema, Mokgadi Malatji

**Affiliations:** 1 Department of Nursing Science, University of Limpopo, South Africa

## Abstract

**Background:**

Absenteeism is a global problem in the working force and this is no exception in the nursing profession. Much attention has been drawn to factors that contribute to absenteeism; however, little attention has been placed on the effects of absenteeism on nurses remaining on duty by their colleagues. Nurses absent themselves leaving behind their colleagues to execute their part of work.

**Objectives:**

To investigate the effects of absenteeism on nurses who remained on duty at a tertiary hospital in Limpopo province.

**Method:**

A quantitative descriptive research approach was chosen to enable the researchers to achieve the research aim. Data collected using structured questionnaires were analysed by descriptive statistics.

**Results:**

The findings indicated that absenteeism has an effect on both the nurses’ psychological and professional well-being, as well as the quality of patient care provided as a result of psychological stress, low morale of nurses and increased workload. The study further revealed the provision of substandard care to patients by those nurses who are remaining on duty, resulting in risk of medical errors that could jeopardise their professional credibility. Therefore, absenteeism creates an unhealthy working environment for nurses remaining on duty.

**Conclusion:**

Nurse managers should provide platforms to address psychological and professional problems experienced by nurses remaining on duty. The study further recommends the introduction of policies that would address absenteeism in the workplace and how nurses who remain on duty could be assisted with the workload of colleagues who continuously absent themselves.

## Introduction

The current worldwide shortage of nurses highlights the importance of understanding the impact and dynamics of absenteeism on relationships between them in healthcare organisations. Absenteeism among nurses has been a centre of attraction in the healthcare system. There are various factors that necessitate nurses’ absenteeism on duty such as absenteeism because of sickness and/or family responsibilities, and as such careful measures need to be taken to reduce the occupational and psychological effects on nurses remaining on duty. The causes of absenteeism among nurses have been the focus of many studies (Alharbi et al. 2016; Vivienne & Bamford 2011), and very little has been done regarding the effect of absenteeism on the nurses remaining at work.

Absenteeism is a global problem that costs countries billions of dollars per year, despite measures and strategies having been formulated to decrease it. This is confirmed by a study conducted by Vivienne and Bamford (2011), stating the factors contributing to people being absent from work. The impact of absenteeism by workers in European healthcare systems has been that of poor patient care satisfaction and increased loss of billions of dollars in the health institutions (Ducklay et al. 2014). In South Africa, absenteeism among nurses is one of the factors causing job dissatisfaction, inevitable increased workload and nurse turnover (Mmamma, Mothiba & Nancy 2015). Therefore, the above authors confirm that absenteeism has an effect on nurses remaining on duty and healthcare institutions. Mudaly and Nkosi (2013) and Baydoun, Dumit and Daouk-Oyry (2016) indicated that increased absenteeism of nurses affects the costs and quality of the health service delivery, staffing of nurses through increased nursing staff shortage and also the productivity of the health institution.

According to Mudaly and Nkosi (2013), nurses absent themselves from work because of personal, professional and organisational conditions. The authors further expressed the effect of absenteeism on nurses remaining on duty to be work-related stress. Gaudine et al. (2011) highlighted that some of the nurses remaining on duty might want to change the workplace in search of better and non-strenuous working environments. In addition, Rantanen (2012) indicated that absenteeism can result in nurses remaining on duty postponing their leave and/or changing the off-duty days in an attempt to cover for the shortage and the increased workload because of unavailability of relevant nurses who at the time are absent. Alharbi et al. (2016) expressed that nurses remaining on duty may suffer from mental problems such as psychological distress, depression and burnout. However, Hong et al. (2012) deliberated on a different view that nurses who are emotionally committed to the health institutions tend to be more productive and are less likely to be absent or quit their jobs.

Absenteeism is on the rise and there is limited intervention to address this problem (Vivienne & Bamford 2011). Interventions that have been in place include addressing employees, concerns, efforts on reducing staff shortages and unimplemented policy on management of absenteeism (Mudaly & Nkosi 2013). Therefore, failure to address absenteeism might render the healthcare institution short-staffed. According to Hong et al. (2012), interventions need to focus on modifying the attitude of nurses who absent themselves from work. Ragani (2012) indicates that most nurses committed to their duties often find themselves confronted by challenges, such as increased workload because of shortage of staff, burnout or fatigue (Gander et al. 2011). Therefore, addressing the absenteeism of nurses will assist in reducing workload, minimising burnout and sustaining nurses’ performance in the healthcare institutions.

## Problem statement

The researcher (M.O.M.), as a professional nurse, observed an increased trend in absenteeism within the hospital wards or units that significantly impacts the healthcare service delivery. As a result, nurses who are committed to their work often find themselves working under strenuous conditions because of inadequate staff. These may lead to poor patient care delivery, feeling overworked and getting sick because of the increased workload. When nurses absent themselves from work, the nurse–patient ratio is affected and this results in the shortage of nurses during the days of absenteeism. The already noted effect of absenteeism on nurses within a healthcare setting is increased workload; however, the researcher believes that there might be more effects of absenteeism especially on the nurses remaining on duty while their colleagues are absent.

## Aim of the study

This study aimed to investigate the effect of absenteeism on nurses remaining on duty using a quantitative research approach.

## Definition of concepts

Effects in this study will mean consequences experienced by nurses remaining on duty at a tertiary hospital of Limpopo province as caused by nurses who stay away from work. Absenteeism refers to when a nurse purposely stays away from work at a tertiary hospital of Limpopo.

## Research method and design

### Research design

A descriptive study design of quantitative research was used in the study. The design assisted the researchers in describing the effects of absenteeism among nurses remaining on duty.

## Material

### Population and sampling

The total population of nurses at one tertiary hospital was 540. The target population, which included all nurses working in the wards, comprised 361 nurses. The total population of all the three categories of nurses was as follows: professional nurses (*n* = 275), enrolled staff nurses (*n* = 140) and enrolled auxiliary nurses (*n* = 125). To ensure equal access to participate in the study, stratified random sampling was used to select nurses from the three categories (professional nurses, enrolled staff nurses and enrolled auxiliary nurses). Respondents were chosen from a computer-generated list provided by the Human Resource Department of the tertiary hospital. Raosoft^®^ sample calculator was used to obtain the sample size for each category of nurses. The sample sizes were as follows: professional nurses (137), enrolled staff nurses (93) and enrolled auxiliary nurses (86). However, because the respondents were given a right to determine their participation status (voluntary), the following nurses participated in the study: 186 respondents which included 62 professional nurses, 62 enrolled staff nurses and 62 enrolled auxiliary nurses. Only nurses who were on day shift were included in the study; the reason was that more absenteeism has been observed among the day shift staff.

### Data collection method

Data were collected through a structured self-developed questionnaire that was personally handed to nurses working in the wards by the researcher (Brink, Van der Walt & Van Rensburg 2012). The researcher was guided by the problems identified and literature to draft the questions regarding the effect of absenteeism on nurses remaining on duty (Guzmán et al. 2016). Validity was ensured by the quantitative experts who checked the questionnaire before data collection. The Cronbach’s alpha indicator ensured reliability with a value of 0.543 (Feldman et al. 2009). The questionnaire was written in English and consisted of two sections: Section A – demographic information, and Section B – effects of absenteeism on nurses remaining on duty. The questions on Section B were based on a two-point Likert scale with values ‘agree’ and ‘disagree’ and it measured ordinal data. Data were collected for 20 working days in 2016.

### Data analysis

Descriptive statistics that included percentages of nurses’ responses according to effects of absenteeism was used to analyse the data (De Vos et al. 2012). Descriptive analysis assisted the researchers in arranging the highest to lowest percentages of effects of absenteeism on nurses remaining on duty. Results were described according to demographic data of nurses and effects of absenteeism on nurses.

### Ethical consideration

Ethical clearance was sought from the Medunsa Research and Ethics Committee (MREC/HS/259/2014). Permission to use a tertiary hospital for conducting the study was sought from the Health Ethics Committee of the Limpopo Provincial Department. Permission to distribute questionnaires among nurses was obtained from the Chief Executive Officer and the nursing manager of a tertiary hospital. The respondents were informed that participation in the study was voluntary and that they can withdraw their participation at any time. The names of the respondents were not captured on the questionnaires to ensure anonymity and each respondent was assigned a number. The respondents signed a consent form as an indication of voluntary acceptance to participate in the study. The researcher recruited the nurses by explaining to them how the study might benefit the healthcare service and/or the hospital.

## Results of the study

The study had 161 (88%) female respondents and 25 (12%) male respondents. Ninety-one (56%) of the female nurses in this study were unmarried, while 70 (43%) were married. The age group of the respondents was mostly between 30 and 39 years (*n* = 91, 49%) and 40 and 49 years (*n* = 58, 30%). The researchers included the demographic data as confirmation from the literature about nursing being dominated by female personnel.

### Increased low morale of nurses in my unit

In this study, 94 (51%) nurses agreed that they had a low morale, with 92 (49%) nurses disagreeing to having low morale, The results indicate that nurses who remained on duty while their colleagues were absent had low morale.

### Interpretation on whether absenteeism increases workload or not

The findings of the study revealed that 103 nurses (55%) agreed with the fact that workload increases because of absent colleagues, while 83 nurses (45%) disagreed that the workload increases when colleagues are absent.

### Feelings of psychological stress resulting from increased workload

The results indicated that 152 nurses (83%) had feelings of psychological stress as a result of increased workload because of their colleagues being absent, while 34 nurses (18%) disagreed with this statement.

### Occurrence of medical errors because of increased workload

The findings revealed that 109 (59%) nurses agreed that they made many medical errors because of the increased pressure to complete their job, while 77 nurses (41%) disagreed with the statement of making medical errors as a result of increased pressure to complete their workload when colleagues are absent.

### Expectations of managers from nurses remaining on duty

The results indicated that 174 nurses (94%) agreed that they are still expected to complete their job with less support from managers, while 12 nurses (6%) disagreed with this fact.

## Discussion of the results

Low morale of nurses remaining on duty can result in higher medico-legal hazards. Nurses with low morale also put the patient’s safety at risk. Ndlhovu (2012) therefore suggests that the morale of nurses remaining on duty should be improved by rewards as a motivating factor. The findings of the study confirmed the effects of absenteeism on nurses remaining on duty as that of increased workload in the wards and consequently reduced quality patient care. The findings further highlighted the effects of absenteeism on patients, especially when nurses’ morale is low, thus affecting the performance of nurses. The study findings further concur with that of Makhado and Davhana-Maselesele (2016) by reporting that nurses with increased workload are prone to burnout, increased job tension and emotional exhaustion.

The findings also indicated that the nurses remaining on duty suffer from psychological problems such as stress and depression. This will affect the staff scheduling in the hospital and result in poor nursing care. A study conducted in Canada indicated stress and tiredness because of the long hours of work as findings for nurses remaining on duty because of absenteeism (Rajbhandary & Basu 2010). Nurses remaining on duty are unable to complete healthcare activities because of the shortage of staff. They are given additional roles because of the absent colleagues and there are more negative incidence reports on days when there is a shortage of nurses as a result of absenteeism (Davey, Cummings & Newburn-Cook 2009; Moret et al. 2012).

Therefore, nurses from these wards are subjected to provide a minimum or no total patient care, especially when their colleagues are absent. This might put the patient’s life at risk, especially with the minimum nursing care and a possible risk of increased morbidity and mortality rate of the patients within the wards. Hennessy (2009) supports the findings by stating that nurses sometimes have little time to attend to patients to give them individualised care because of the shortage of staff. The findings are similar to those of Madibana (2010) and Moret et al. (2012), who indicated that there is a reduction of quality patient care rendered by nurses when they are absent. Nurses often experience difficulty in meeting patients’ needs, especially when covering for specialised absent colleagues. The findings further indicate that, despite the increased workload in the wards, low morale of nurses remaining on duty and minimum provision of patient care, nursing managers are not supporting the nurses remaining on duty. Lack of support from the nurse managers subjects the nursing staff to low morale and possible poor performance when providing nursing care services. These findings are supported by Mudaly (2009) and Indris et al. (2012), who indicated that nurse managers experience pressure when there is absenteeism in their unit and provide poor support to their subordinates.

## Practical implications

Determining the effects of absenteeism on nurses remaining on duty is significant for efficiency and effectiveness of the healthcare institutions. Management of absenteeism, which includes determining the causes of absenteeism, recording the trends of nurses’ absenteeism and implementing the absenteeism management policy, is a key priority of the nurse managers in healthcare institutions. Because nurses play an important role in patient care, assessing and managing both personal and family concerns is significant to reduce absenteeism at workplace. Nurses’ absenteeism results in low staff morale, increased shortage of nurses and workload. Nurses with low morale have substandard performance in the healthcare institutions, thus resulting in increased infectious diseases, increased morbidity and mortality rates, and possible conflicts among each other (Gaudine & Gregory 2010). The nurses remaining on duty because of absenteeism might perceive the working environment unsafe and possibly consider getting another job outside the healthcare institution. High staff turnover from the nurses might increase because of minimum support by the nurse managers. Nurses’ absenteeism affects patient care and as such patients might experience prolonged hospitalisation as a result of high workload and low morale of the nurses remaining on duty. The healthcare institution might experience less productivity as a result of poor performing nurses. The healthcare institutions might also have financial implication costs, especially with regard to hiring replacement nurses.

## Limitations

This study was conducted at one tertiary hospital in Limpopo province and therefore cannot be generalised to other healthcare institutions of different provinces.

## Recommendations

Nurse managers should provide platforms to address psychological and professional problems experienced by nurses remaining on duty. The existing wellness programme could be enhanced to assist nurses in coping with psychological and professional problems. The study further recommends the introduction of policies that would address absenteeism in the workplace and how nurses who remain on duty could be assisted with the workload of colleagues who continuously absent themselves. Strict monitoring of nurses absenteeism by managers should be employed to address this anomaly.

## Conclusion

The study concludes that absenteeism creates a burden for nurses who remain on duty because they have to cover for themselves and for those colleagues who are absent. In addition, absenteeism causes an unhealthy working environment among nurses remaining on duty because they experience psychological stress which affects how they execute their professional expectations. The study indicated that quality total patient care is not implemented because of increased workload, shortage of nurses and decreased nurses’ morale.
